# Observations-Based Approach to the Estimation of the Oxidizing Capacity of the Atmosphere of Central London over the 2000–2024 Period

**DOI:** 10.1021/acs.jpca.6c02946

**Published:** 2026-09-01

**Authors:** M. Anwar H. Khan, Richard G. Derwent, Frank A. F. Winiberg, Carl J. Percival, Dudley E. Shallcross

**Affiliations:** † Atmospheric Chemistry Research Group, School of Chemistry, 1980 University of Bristol, Cantock’s Close, Bristol BS8 1TS, U.K.; ‡ rdscientific, Newbury, Berkshire RG14 6LH, U.K.; § Jet Propulsion Laboratory, California Institute of Technology, 4800 Oak Grove Dr, Pasadena, California 91109, United States; ∥ Department of Chemistry, University of Western Cape, Robert Sobukwe Road, Bellville 7535, South Africa

## Abstract

An observations-based approach has been applied to the air quality monitoring data at the London Marylebone Road over the 2000–2024 study period to estimate the oxidizing capacity of the atmosphere in Central London. The mean of the daylight hours-averaged OH number densities is found to be 4.0 × 10^6^ molecules cm^–3^, a value which is consistent with the OH concentrations measured at different polluted sites in different campaigns. A strong seasonal cycle of OH number density is observed with a peak in summer and a trough in winter. Although there are large changes in emissions from traffic and other sources and in ambient ozone levels in recent years, the nonlinear regression fitted seasonal cycle through the 24 years of daylight hours-averaged OH number densities exhibits a slight but highly statistically significant downward trend of (0.011 ± 0.0026) × 10^6^ molecules cm^–3^, (0.3 ± 0.07) % per decade (where the quoted confidence intervals represent 1 – σ and the 2 – σ confidence limits do not encompass zero). The concept of OH collapses is introduced in the study and gives an idea about the impacts of air quality changes on the oxidizing capacity of the London atmosphere. The rise in the number of OH collapses during the early years of the study period shown is driven by the increased direct emissions of NO_2_ from diesel-engined vehicles. The implication of a collapse should be further researched; analysis could be undertaken on a shorter than hourly time scale to assess the impact on OH collapse on a scale more comparable to its lifetime. It should be noted that no long-term measurements of HCHO or HONO were available, two key components of the OH budget, and these have been estimated as described in the text.

## Graphical abstract

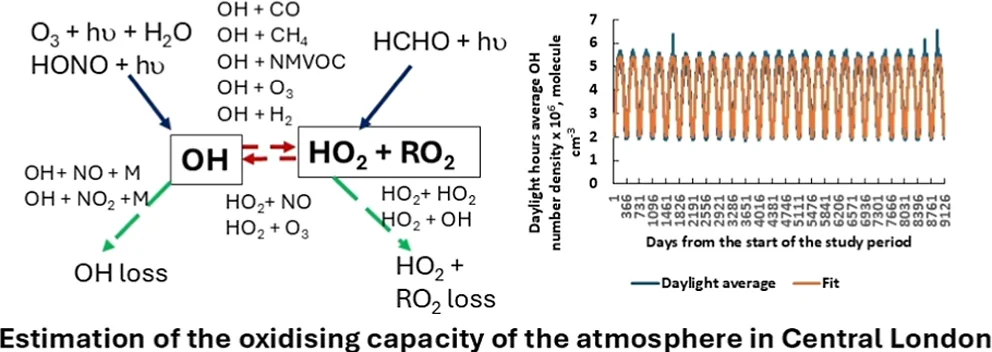

## 1 Introduction

The hydroxyl radical (OH) is a species produced in the troposphere that acts like a chemical detergent, initiating the removal of the pollutants and thereby controlling the oxidizing power of the atmosphere. The ability to accurately determine the current and future concentrations of OH, and hence the atmospheric oxidative capacity (AOC), is therefore vital in developing future emission controls that will be effective in the prevention of both primary and secondary pollution. The studies^1,2^ have shown that our understanding of the chemistry of OH radicals remains a challenge with OH interferences found from some measurement techniques. In addition, over the years, rate coefficient values for the formation and loss processes utilized in atmospheric models have been revised, e.g., the reaction between OH and NO_2_.^3^

The measurement of OH *in situ* has proven to be challenging because of its high reactivity, short lifetime, and low tropospheric concentrations.^4^ Some techniques such as laser-induced fluorescence (LIF)-fluorescence assay by gas expansion (FAGE), chemical ionization mass spectrometry (CIMS), or differential optical absorption spectroscopy (DOAS) are used to measure OH levels in the atmosphere which could have interferences yielding accuracies for OH concentration measurements within 10%, 16%, and 7%, respectively.^5^ A recent study^1^ reanalyzed OH measurements from previous measurement campaigns and noted that data using a ground-based tropospheric hydrogen-oxides sensor from Penn State and a photofragmentation/laser-induced fluorescence FAGE used by Indiana University overestimated OH, and when reanalyzed, their data were consistent with other studies. Laser flash photolysis coupled to laser-induced fluorescence (LFP-LIF) can measure total OH reactivity by producing OH in isolation via the photolysis of O_3_, which makes it a much more suitable method for urban areas with high NO_*x*_ concentrations.^6^ Nevertheless, measurement data are often not available on a frequent enough time scale, and achieving global coverage, including all remote regions, is still a significant barrier to obtaining easy-to-access high-resolution data of OH concentrations.^7^ The current difficulties in quantifying OH concentrations and repercussions of the limited understanding of the missing OH reactivity have been exemplified numerous times, including during the ClearfLo study^8^ and the Chinese megacity study.^9^

Due to the reactivity, and hence short lifetime, the atmospheric OH concentration is not commonly measured over long time periods, necessitating its calculation from the rates of OH radical production and loss.^4,10^ In this study, we have adopted an observations-based approach and employed a daylight hours-averaged OH concentration metric to distinguish our methodology from empirical air pollution modeling based on the current understanding of air pollutant emissions and atmospheric processes. The observations-based approach to the estimation of the oxidizing capacity of the atmosphere in Central London is applied here to the air quality data monitored at the Marylebone Road roadside location in Central London over the 2000–2024 study period, to yield an estimate for the OH number density for each daylight hour for each day of the 25 year study period. This analysis will help to evaluate the impact of varying emission compositions and volumes on the OH concentrations and therefore attempt to understand the impact of likely future changes in the emissions. For example, in 2020, the emissions caused by reduced vehicular traffic from the COVID-19 lockdowns resulted in a dramatic increase in oxidative chemistry during that year.^11–13^

In addition to this, an exploration into the number of OH collapses defined in this study as a reduction in its concentration by two standard deviations from the mean has been undertaken. Collapses in OH have not yet been a considerable focus in atmospheric research, with few published studies being carried out into the prevalence and possible significance of such occurrences on the atmosphere and subsequently human health. Therefore, this study will attempt to quantify the number of collapses experienced in London Marylebone Road and to identify whether this is an area suitable for future research.

## 2 Methodology

Jeffries and Tonnesen^14^ described how to analyze the fast photochemistry in any reaction system with a view to understanding how atmospheric chemistry works at the chemical process level. The fast photochemistry of the atmosphere of London involves two main free radical species: the hydroxyl (OH) radical and the peroxy radical (HO_2_ + RO_2_) pool and six main interconnecting reaction processes (see Figure 1).

**Figure 1:**
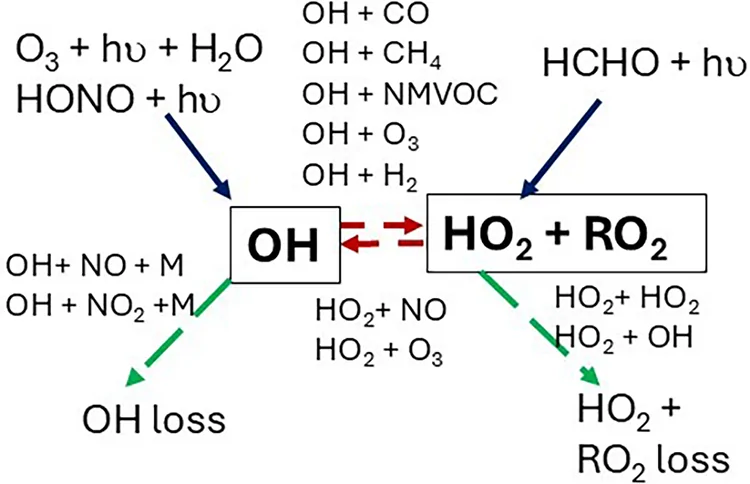
Main possible reactions of the fast photochemical balance for London’s atmosphere. The OH and HO_2_ radical production fluxes are represented with solid blue arrows, the radical recycling interconversion fluxes with pecked red arrows and the OH and HO_2_ radical sinks with green dashed arrows.

### 2.1 Direct OH Sources

O_3_ photolysis is a direct OH source through

$$\begin{array}{cc} O_{3}+h\nu \to O^{1}D+O_{2} & J_{1}\\ O^{1}D+O_{2}\to O^{3}P+O_{2} & k_{1}\\ O^{1}D+N_{2}\to O^{3}P+N_{2} & k_{2}\\ O^{1}D+H_{2}O\to OH+OH & k_{3} \end{array}$$

where fraction of ozone producing OH defined by a factor

1 $$\alpha =k_{3}\left[H_{2}O\right]/\left(k_{1}{\left[O_{2}\right]+k}_{2}{\left[N_{2}\right]+k}_{3}\left[H_{2}O\right]\right)$$

HONO photolysis is another OH source through

$$\begin{array}{cc} HONO+h\nu =OH+NO & J_{2} \end{array}$$

2 $$Total\;direct\;OH\;source=\alpha J_{1}\left[O_{3}\right]+J_{2}\left[HONO\right]$$

### 2.2 OH to Peroxy Radical Interconversion Sinks

The most important term in the OH to peroxy radical interconversion sinks is the OH + CO reaction:

$$\begin{array}{cc} OH+CO\to H+{CO}_{2} & k_{4} \end{array}$$

which is rapidly followed by

$$H+O_{2}+M\to {HO}_{2}+M\;\left(assumed\;instantaneous\right)$$

The reactions of OH with the other species, e.g., methane (CH_4_); nonmethane volatile organic compounds (NMVOCs) including aldehydes, ketones, alkanes, and alkenes; and aromatic compounds, hydrogen (H_2_) and ozone (O_3_), can also be involved in the OH to peroxy radical interconversion sinks.

### 2.3 Direct HO_2_ Sources

HCHO photolysis occurs by two routes, one of which is a direct source of HO_2_ radicals:

$$\begin{array}{cc} HCHO+h\nu \to H+HCO & J_{3}\\ HCHO+h\nu \to H_{2}+CO & J_{4} \end{array}$$

$$H+O_{2}+M\to {HO}_{2}+M\;\left(assumed\;instantaneous\right)$$

$$HCO+O_{2}\to {HO}_{2}+CO\;\left(assumed\;instantaneous\right)$$

3 $$Total\;direct\;{HO}_{2}\;sources=J_{3}\left[HCHO\right]\times 2$$

### 2.4 HO_2_ to OH Interconversion Sources

These involve the HO_2_ + NO and HO_2_ + O_3_ reactions.

$$\begin{array}{cc} {HO}_{2}+NO\to OH+{NO}_{2} & k_{5}\\ {HO}_{2}+O_{3}\to OH+{2O}_{2} & k_{6} \end{array}$$

### 2.5 Direct OH Sinks

This is largely the OH + NO_2_ + M and OH + NO + M reactions.

$$\begin{array}{cc} OH+{NO}_{2}+M\to {HNO}_{3}+M & k_{7}\\ OH+NO+M\to HONO+M & k_{8} \end{array}$$

$$Total\;direct\;OH\;sinks=k_{7}{\left[OH\right][NO}_{2}{]\left[M\right]+k}_{8}\left[OH\right]\left[NO\right]\left[M\right]$$

### 2.6 Calculation of OH Steady State

By assuming that [OH] and [HO_2_] are in steady state and substituting [HO_2_] in the steady-state expression for [OH], we obtain

4 $$Direct\;OH\;source+recycled\;{HO}_{2}\;sink=direct\;OH\;sink+recycled\;OH\;sink$$

5 $$Direct\;{HO}_{2}\;source+recycled\;OH\;sink=direct\;{HO}_{2}\;sink+recycled\;{HO}_{2}\;sink$$

As HO_2_ + HO_2_ is unimportant in the high NO_*x*_ conditions of the atmosphere of London, we can consider that the direct HO_2_ sink = 0, which gives

6 $$Recycled\;{HO}_{2}\;sink=direct\;{HO}_{2}\;source+recycled\;OH\;sink$$

Substituting this into (eq 4) gives

7 $$Direct\;OH\;source+direct\;{HO}_{2}\;source=direct\;OH\;sink$$

$$\alpha J_{1}\left[O_{3}\right]+J_{2}\left[HONO\right]+J_{3}\left[HCHO\right]=k_{7}{\left[OH\right][NO}_{2}{]+k}_{8}\left[OH\right]\left[NO\right]$$

8 $$\left[OH\right]=\frac{\alpha J_{1}\left[O_{3}\right]+J_{2}\left[HONO\right]+J_{3}\left[HCHO\right]}{k_{8}\left[NO\right]\left[M\right]+k_{7}\left[NO_{2}\right]\left[M\right]}$$

where α is calculated using eq 1. The photolysis constants, J_1_, J_2_, and J_3_, are calculated using the photolysis parameters set out by the Master Chemical Mechanism, MCM (https://www.mcm.york.ac.uk/MCM/rates/photolysis). The parameters for the complex rate coefficients, *k*_7_ and *k*_8_, are taken from DeMore et al.^15^ and Woods et al.,^16^ respectively. Ground-level hourly measurement data of O_3_, NO, and NO_2_ were obtained from the Department for Environment, Food and Rural Affairs (DEFRA) as part of the Automatic Urban and Rural Monitoring Network (AURN) site in London Marylebone Road as it measures the most comprehensive set of VOCs for an urban site in the UK (https://uk-air.defra.gov.uk/networks/network-info?view=aurn).

HONO measurement data in Central London are not available as they are not included as part of the DEFRA network. Because of the importance of this trace gas as a direct OH source, we estimated [HONO] using DEFRA hourly NO_*x*_ data from London Marylebone Road. The hourly NO_2_ data for the period of 2000–2024 multiplied by the diurnal HONO/NO_*x*_ ratios reported by Lee et al.^17^ for North Kensington, London, as part of the Clean air for London (ClearfLo) project. HCHO measurement data in Central London are also not available. The assumption was therefore made that HCHO is imported into the London atmosphere from the background troposphere. We estimate the northern hemisphere tropospheric background HCHO from its main source, OH oxidation of methane, and its photochemical sinks. We took [OH] = 1 × 10^6^ molecules cm^–3^ because this number is close to the typical northern hemisphere tropospheric 24 h mean concentration.^18^ There is of course an underestimation for HCHO in our calculation as sources other than methane exist, and we present an estimate of this underestimation in the Supporting Information. The calculation in the Supporting Information estimates that the underestimation is of the order of around 35% in London, mainly due to methanol and isoprene concentrations being additional contributors.

By assuming the HCHO is formed only from the oxidation of CH_4_ and in a steady-state, the concentration of HCHO is calculated using eq 9.

9 $$\left[HCHO\right]=k_{9}{[CH}_{4}]\left[OH\right]/\left(J_{3}{+J}_{4}{+k}_{10}\left[OH\right]\right)$$

where the rate constant of the reaction of CH_4_ with OH (*k*_9_) and HCHO with (*k*_10_) and the photolysis parameters for the photolysis of HCHO (J_3_ and J_4_) are obtained from MCM.

As the oxidation of CH_4_ is the main source of HCHO, we used estimated CH_4_ mixing ratios as a constant hourly measurement calculated from a monthly average for the Greater London Area.^19^ The globally averaged monthly mean CH_4_ concentrations for 2000–2024 were collected from the Global Monitoring Division of NOAA’s Earth System Research Laboratory.^20^ Saboya et al.^21^ reported monthly CH_4_ concentrations at Imperial College London in central London (2.3 miles far from London Marylebone Road) for the period of March 2018 to October 2020. A conversion factor was derived by dividing the Imperial College London CH_4_ concentrations by NOAA’s global CH_4_ concentration, which was then multiplied with the globally averaged monthly mean CH_4_ concentrations for 2000 to 2024.

The years of 2000 to 2024 were studied to allow analysis of how pollution has changed over the last two decades and to assess the oxidative capacity of the atmosphere by investigating the change of OH trends and the number of OH collapses.

## 3 Results and Discussion

### 3.1 OH Trends

Daylight-averaged OH number densities were calculated for all the days during the study period of 2000–2024 using the observations-based approach and eq 8. The OH number densities were averaged over the daylight hours in each day, and the daylight hours-averaged OH number densities are presented in Figure 2.

**Figure 2:**
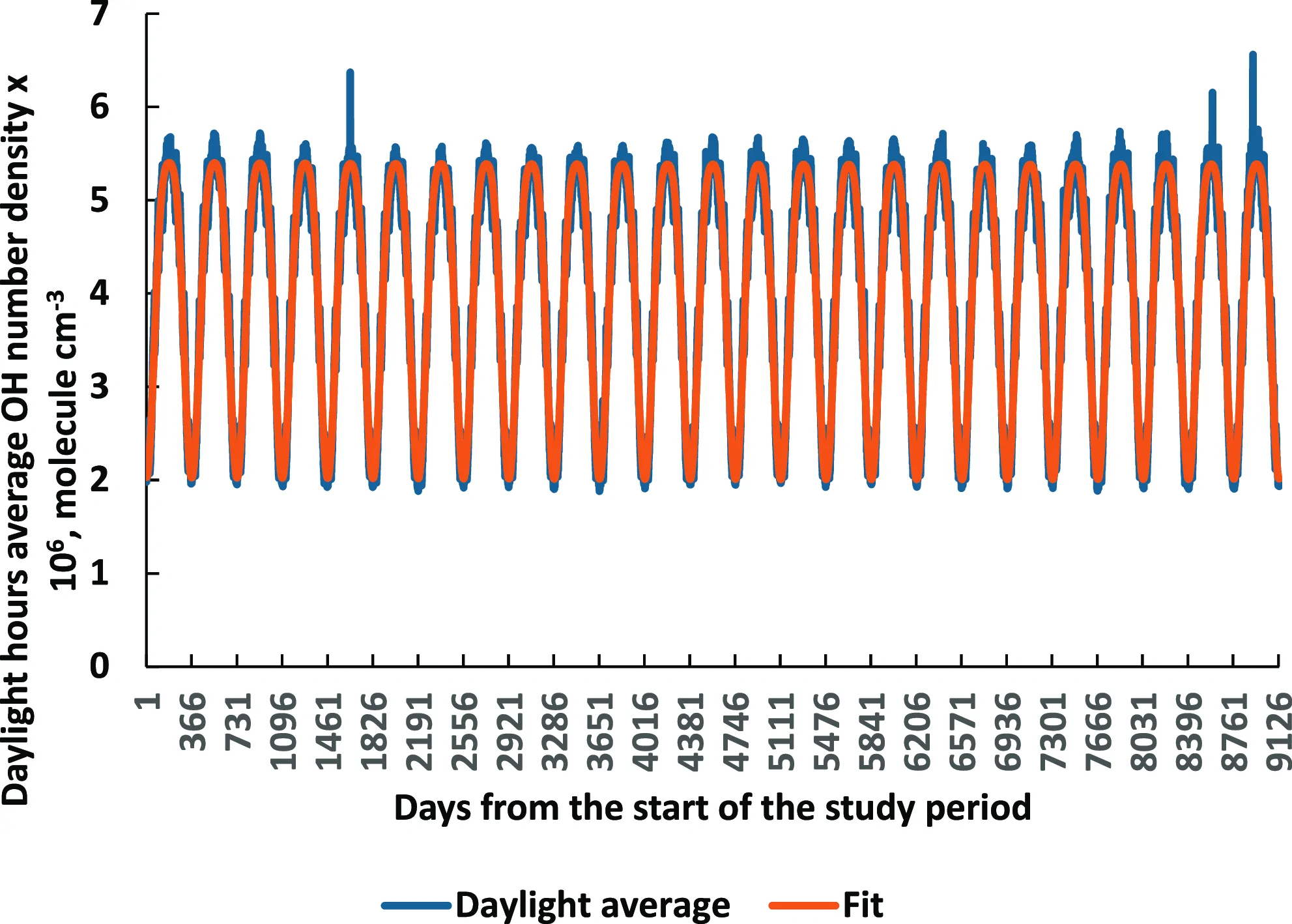
Time series of the estimated and fitted daylight hours-averaged OH radical number densities from 1st January 2000 to 31st December 2024.

The mean of the daylight hours-averaged OH number densities was found to be 4.0 × 10^6^ molecules cm^–3^ with a maximum of 6.6 × 10^6^ molecules cm^–3^ observed on 2 June 2024 and a minimum of 1.9 × 10^6^ molecules cm^–3^ observed on 31 December 2009. Our results are consistent with a recent observation-based method (OBM) analysis of OH concentrations (4.2 × 10^6^ molecules cm^–3^) for Europe during 2000–2023.^22^ Wei et al.^22^ showed an increasing trend of OH levels for most of Europe for the period of 2000–2023; however, inspection of Figure 2d from their work suggests a decreasing trend for the UK, in agreement with the analysis from this work. The results are also comparable with the measurement data from the different environmental sites at different seasons of the study period, 2000–2024 (Table 1). Overall, our results are found to be within the range of the literature data reported for different field campaigns with polluted environments (e.g., PUMA, TEXAQS, PMTACS, MCMA, TORCH, IMPACT, MILAGRO, TRAMP, and AIRPRO) (Table 1). However, the short OH tropospheric lifetime resulted in the large spatial and temporal OH variations so that the average OH number densities reported here are not necessarily the ideal method of comparison with the measured data from the different campaigns. London Marylebone Road site will have a specific pollution composition, solar zenith angle, and temperature which will impact the rate of OH production and loss. Therefore, any disparity with the literature data in Table 1 is unlikely to be suggestive of a significant uncertainty due to missing potential sources and sinks in the method of OH calculation in this study. The daylight hours-averaged OH number densities exhibited a marked seasonal cycle (Figure 3), with a summertime maximum with an average of 5.3 × 10^6^ molecules cm^–3^ and a wintertime minimum with an average of 2.4 × 10^6^ molecules cm^–3^ due to the variability of the photolysis of O_3_ and HONO in different seasons. The weather conditions are known to significantly affect HONO and O_3_ formation, with high temperatures and low humidity and low wind speed favoring their formation during summer months, which in turn boosts the production of OH.^23^

**Table 1:** Summary of Spatial and Temporal OH Measurements at Different Environmental Sites for the Period 2000–2024

campaign	time	location	OH concentration (×10^6^ molecules cm^–3^)	references
PUMA-2	Jan-Feb 2000	Birmingham, UK (53 °N, 2 °W)	Midday average 1.5	Heard et al.^24^
HOPE	Jun-Jul 2000	MOHp, Germany (48°N, 11°E)	Max midday 4.5–7.4	Handisides et al.^25^
TEXAQS	Aug-Sep 2000	Houston, Texas, USA, (29°N, 95°W)	Midday maximum average 19.7	Mao et al.^26^
PMTACS-1	Jun-Aug 2001	Whiteface mountain, New York (41 °N, 74 °W)	Daytime maximum 5–20	Ren et al.^27^
MINOS	Aug 2001	Crete (35 °N, 26 °E)	Daytime average 4.5	Berresheim et al.^28^
PMTACS-2	Jun-Aug 2002	Whiteface mountain, New York (41 °N, 74 °W)	Average noontime Max 2.6	Ren et al.^29^
NAMBLEX	Jul-Sep 02	Mace Head, Ireland (53°N, 10°W)	3–8 at noon 24 h average 0.91	Smith et al.^30^
Rock Springs	May-Jun 02	Pennsylvania, USA (39 °N, 77 °W)	Daytime average 3.9	Ren et al.^31^
RISFEX	Sep 03	Rishiri Island, Japan (45 °N, 141 °E)	Average Max 2.7	Kanaya et al.^32^
MCMA	Apr 03	Mexico City (19 °N, 99 °W)	Max daytime 8–13	Shirley et al.^33^
TORCH	Jul-Aug 03	Writtle, Essex, UK, (51°N, 0°E)	Daytime 1.2–7.5	Lee et al.^34^
IMPACT IV	Jan-Feb 04	Tokyo, Japan (35°N, 139°E)	Median daytime peak 1.5	Kanaya et al.^35^
PMTACS-3	Jan-Feb 04	Whiteface mountain, New York (41 °N, 74 °W)	Maximum average 1.4	Ren et al.^36^
IMPACT L	Jul-Aug 04	Tokyo, Japan (35°N, 139°E)	Median daytime peak 6.3	Kanaya et al.^35^
CHABLIS	Jan-Feb 05	Halley Base, Antarctica (75 °S, 26 °W)	Average Max 7.9 Average daytime 3.9	Bloss et al.^37^
HO_*x*_Comp	Jul 05	Julich, Germany (51 °N, 6 °E)	5–15 during noon	Kanaya et al.^38^
MILGARO	Mar 06	Mexico City (19 °N, 99 °W)	Max daytime 2–15	Dusanter et al.^39^
PRIDE-PRD	Jul 06	Guangzhou, Southern China (25 °N, 113 °E)	Average noontime 15	Hofzumahaus et al.^40^
TRAMP	Aug-Sep 06	Houston, Texas, USA (29°N, 95°W)	Midday maximum average 14.8	Mao et al.^26^
RHaMBLe	May-Jun 07	Cape Verde (17 °N, 25 °W)	Midday Max 9	Whalley et al.^41^
BEARPEX-1	Aug-Oct 07	Blodgett forest, California, (39°N, 120°W)	Noontime average 7	Wolfe et al.^42^
COBRA	Feb-Mar 08	Kuujjuarapik, Canada (55 °N, 78 °W)	Average noontime 0.77 and max 1.16	Edwards et al.^43^
OP3	Apr-May 08	Danum Valley (5 °N, 118 °E)	Noontime average 2 Daytime average 1.2	Whalley et al.^44^
BEARPEX-2	Jun-Jul 09	Blodgett forest, California, (39°N, 120°W)	Noontime average 4 (wave), 1.5 (chem)	Mao et al.^45^
OOMPH	Mar 07	Atlantic Ocean (28–57°S, 46°W-34 °E)	Max 6	Hosaynali Beygi et al.^46^
DOMINO	Nov-Dec 08	El Arenosillo, southern Spain (37 °N, 7°W)	Max 4 in continental air and max 2.5 in oceanic air	Sinha et al.^47^
SOS	Feb-Mar, Jun, Sep 09	Cape Verde (17°N, 25°W)	Midday Max 9	Vaughan et al.^48^
ClearfLo	Jul-Aug 12	North Kensington, London (51°N, 0.12°W)	Max daytime 4.9	Whalley et al.^8^
ICOZA	Jun-Jul 15	Weybourne, Norfolk, UK (53°N, 1°E)	Max daytime 2.6–17	Woodward-Massey et al.^49^
AIRPRO	May-Jun 17	Central Beijing (40°N, 116°E)	Max 28 afternoon	Whalley et al.^50^

**Figure 3:**
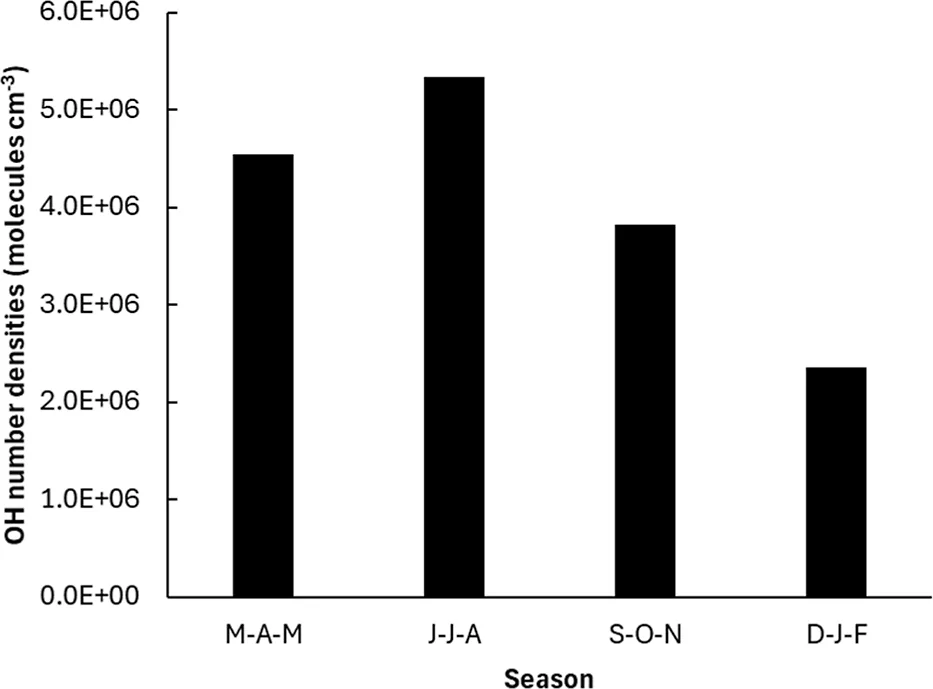
Seasonal variation of daylight hours-averaged OH radical number densities for the time period of 1st January 2000 to 31st December 2024.

Overall, this seasonal cycle was found to be highly stable, with little change in either the timing or magnitudes of the seasonal cycles observed in each year of the 25 year study period (Figure 2).

### 3.2 Analysis of Trends with Time and Season

An expression involving three polynomial terms and two sine terms of the form (eq 10)­

10 $$\left[OH\right]=a+bx+cx^{2}+a_{1}\sin\left(x+\varphi_{1}\right)+a_{2}\sin\left(2x+\varphi_{2}\right)$$

was fitted through the time series of daylight hours-averaged OH number densities, [OH], using nonlinear regression software.^51^ In this equation, time is represented by x, where x increases continuously by 2π radians each year. The fitted equation accounted for over 97% of the variance in the daylight hours-averaged OH, and the 2 – σ confidence intervals of the seven fitted parameters, a, b, c, a_1_, a_2_, φ_1_, and φ_2_, did not encompass zero and hence were statistically significant. The two sine terms represent a concise description of the seasonal cycle in the daylight hours-averaged OH. The time variable x was set to zero at the start of the time series of observations on first January 2000. The fitted seasonal cycle and the observations are compared in Figure 2, which shows that they line up exactly across the entire study period.

The values of the seven fitted parameters and their 2 – σ confidence limits are given below:

$$\begin{array}{l} a,4.04\pm 0.006\times 10^{6}\;molecule\;{cm}^{-3}\\ b,-0.0026\pm 0.0011\times 10^{6}\;molecule\;{cm}^{-3}\;{year}^{-1}\\ c,78\pm 44\;molecule\;{cm}^{-3}\;{year}^{-2}\\ a_{1},1.687\pm 0.003\times 10^{6}\;molecule\;{cm}^{-3}\\ a_{2},-0.327\pm 0.003\times 10^{6}\;molecule\;{cm}^{-3}\\ b_{1},-1.59\pm 0.002,radian\\ b_{2},1.531\pm 0.009,radian \end{array}$$

Monte Carlo sampling was employed to determine the dates of the seasonal maxima and minima, revealing a minimum in the fitted seasonal cycle at 1 January and a maximum at 3 July. It appeared, therefore, that the fitted seasonal cycle closely followed the annual cycle in solar radiation at the London Marylebone Road monitoring station.

The fitted seasonal cycle through the 24 years of daylight hours-averaged OH number densities exhibits a slight but highly statistically significant (because the 2 – σ confidence limits do not encompass zero) downward trend of (0.011 ± 0.0026) × 10^6^ molecules cm^–3^ decade^–1^ or −(0.3 ± 0.07)% per decade. This is in marked contrast to the annual mean air quality data which show large trends over the 2000–2024 period. Ozone, for example, exhibits a close to a factor of 2 to 3 increase from 13.2 ppb (in 2020) to 32.2 ppb (in 2024) at the London Marylebone Road monitoring station over the study period. The contribution of ozone photolysis to OH number densities is found to be significantly lower (∼1–2%) than that of HONO photolysis, which is consistent with the study of Clean air for London project (ClearfLo).^8^ The recent increases in O_3_ concentrations in London Marylebone Road enhance the contribution of ozone photolysis to OH number densities from 1.7 × 10^5^ molecules cm^–3^ s^–1^ (in 2000) to 3.2 × 10^5^ molecules cm^–3^ s^–1^ (in 2024) but have insignificant impact on the total OH number densities. The significant decreases in NO_*x*_ concentrations in London Marylebone Road from 151.8 ppb (in 2000) to 30.2 ppb (in 2024) decrease the formation of HONO resulting in the reduction of contribution of HONO photolysis to OH number densities from 2.0 × 10^8^ molecules cm^–3^ s^–1^ to 3.6 × 10^7^ molecules cm^–3^ s^–1^.

Figure 4a and b present the annual mean NO_*x*_ and NO_2_ concentrations at 23 roadside and kerbside sites in the London Air Quality Network (LAQN) (https://londonair.org.uk) expressed relative to their respective 2006 value = 1.0. These plots show how the NO_*x*_ and NO_2_ levels have been declining across the most polluted LAQN sites in the years before our study period began due to the installation of three-way exhaust catalysts in petrol-engined motor vehicles. The impact has been much stronger in annual mean relative NO_*x*_ levels compared with annual mean relative NO_2_ levels, the latter having remained largely constant from 2000 onward.

**Figure 4:**
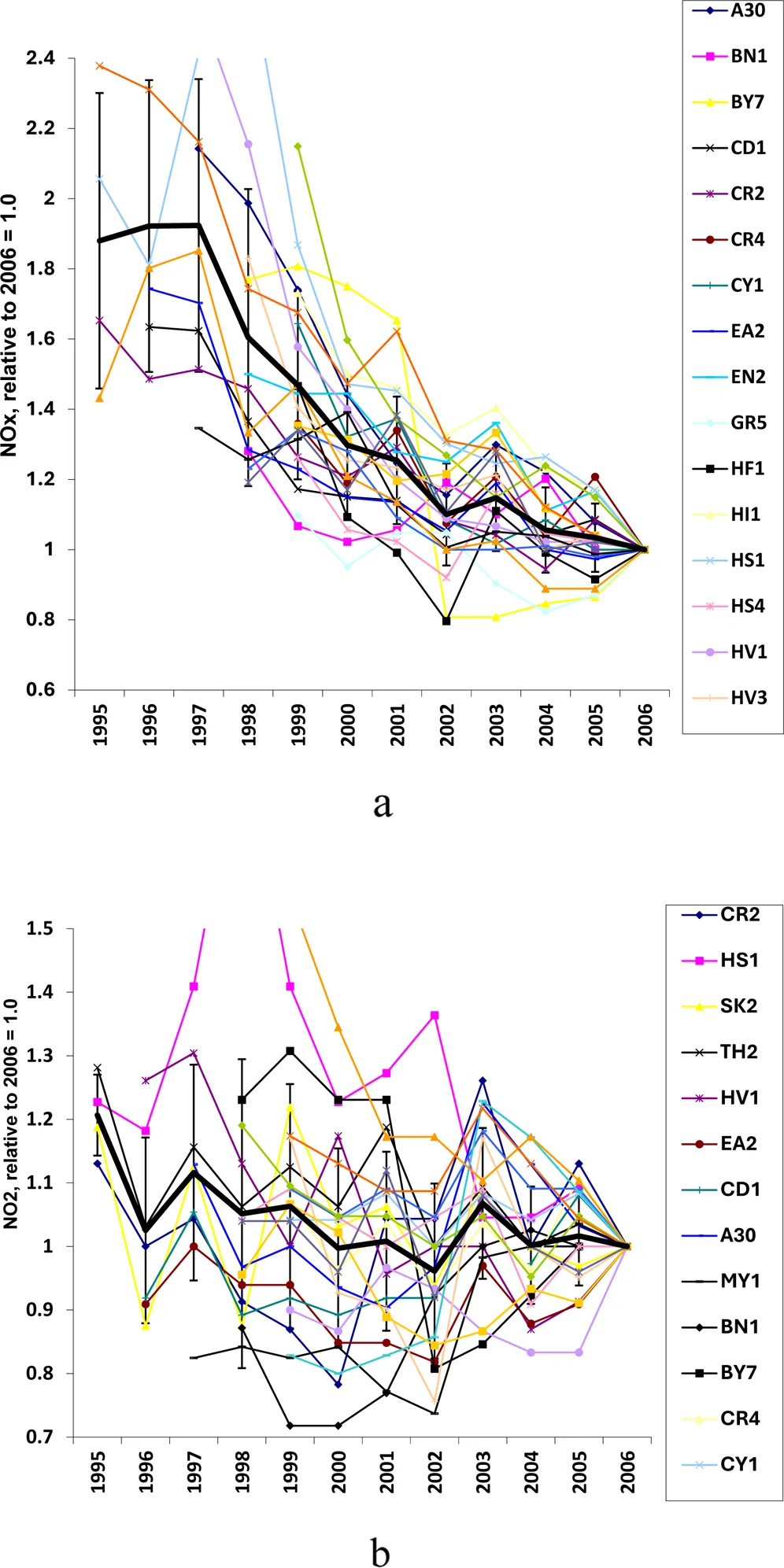
(a) Time series of the annual mean NO_*x*_ concentrations relative to 2006 = 1.0 at the roadside and kerbside sites in the LAQN over the period from 1995 to 2006 leading up to the study period at the London Marylebone Road (MY1) roadside site. (b) Time series of the annual mean NO_2_ concentrations relative to 2006 = 1.0 at the roadside and kerbside sites in the LAQN over the period from 1995 to 2006, leading up to the study period at the London Marylebone Road (MY1) roadside site.

Focusing on the early years of the 25 year study period (2000–2006), the decline in annual mean relative NO_*x*_ levels has been about 4% per year, and, in contrast, annual mean relative NO_2_ levels have remained roughly constant. If the annual average oxidizing capacity of the heavily polluted atmosphere at London Marylebone Road was dependent solely on the [NO_*x*_]/[NO_2_] ratio because the HONO source of OH is related directly to the levels of NO_*x*_ and the OH + NO_2_ sink is related directly to the level of NO_2_, there should have been a decline of about −4% per year in the atmospheric oxidizing capacity, over the 2000–2006 period, driven by the strong trend in [NO_*x*_]. This decline in the average oxidizing capacity of about −4% per year was not seen in the estimates as revealed by the nonlinear regression fitting procedure described above which generated a trend of (0.3 ± 0.07) % per decade. The atmospheric oxidizing capacity is therefore not solely controlled by NO_*x*_ and NO_2_ alone and there must be other factors in control, presumably a strong influence from HCHO photolysis. Unfortunately, HCHO is not routinely monitored in the UK.

### 3.3 OH Collapses

In this section, the concern is not with the majority of the observed daylight hours-averaged values that are fitted well by the seasonal cycle defined by the seven parameters noted earlier but with the tiny minority of daylight hours-averaged points that fall well below the fitted curve. Figure 5 presents the differences between the observed daylight hours-averaged OH number densities and the fitted curve for each day of the study period. There are a larger number of days when the differences are negative (observations less than the fit) compared with when the differences are positive (observations larger than the fit). Not only are there a larger number of days when the differences are negative but these days show much larger differences. Indeed, there are a significant number of days, 98 in total, when the observations–fit differences are greater than two standard deviations from the mean. This is illustrated in Figure 5, where the blue line shows the approximate position of the points that are two standard deviations from the mean. The largest differences from the mean of over −0.57 × 10^6^ molecules cm^–3^, approaching three standard deviations from the mean, are highlighted in Figure 5 on 25th August 2019. The NO_2_ levels on 25 August 2019 are found to be 95 ppb, which is 1.5 order higher than the yearly average of NO_2_ levels (62 ppb) during 2019. It is possible that the NO_2_ fraction of NO_*x*_ is driving the OH collapse.

**Figure 5:**
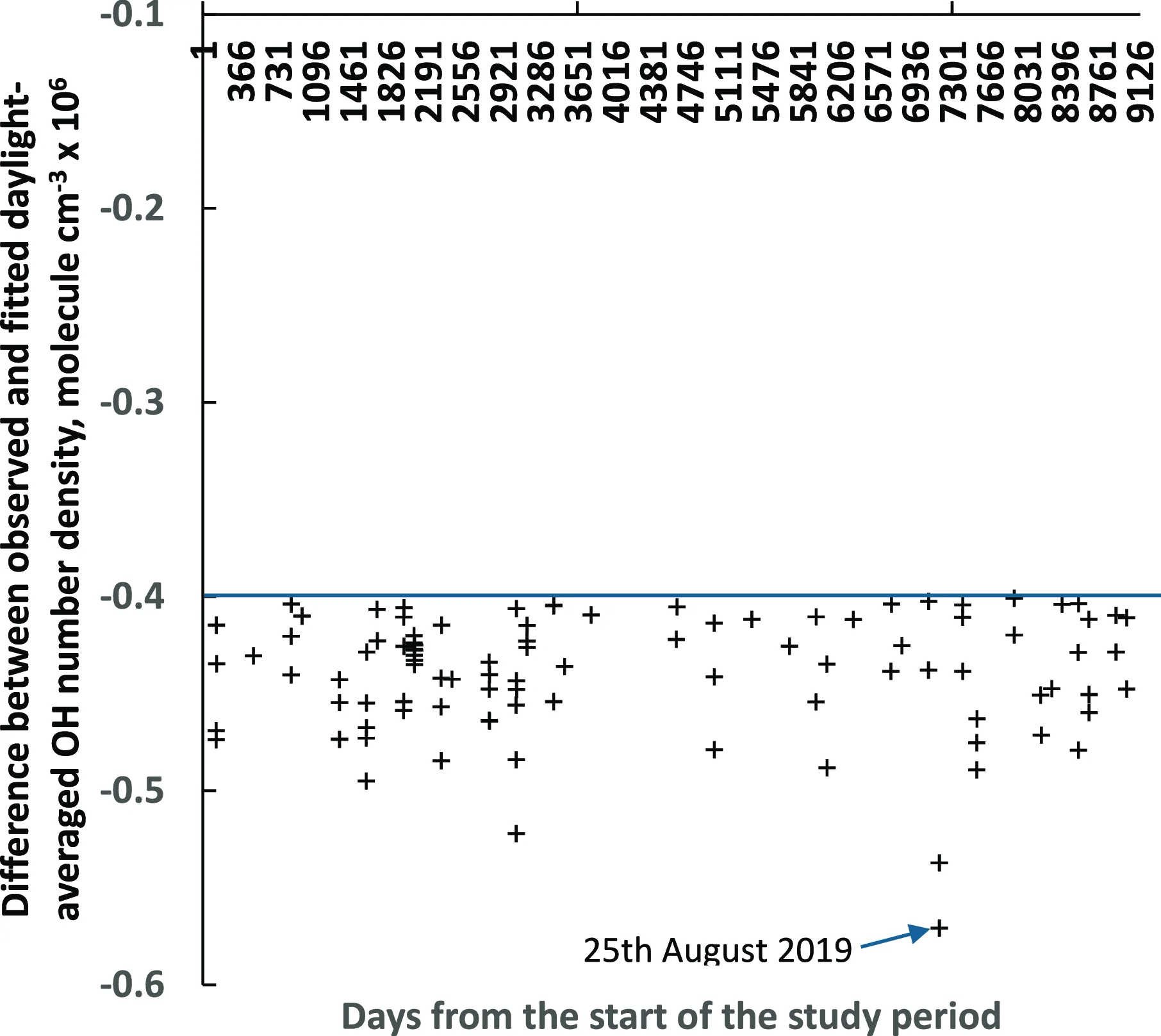
Difference between the observed and fitted daylight-averaged OH number density from 1st January 2000 to 31st December 2024. The blue line shows the position of two standard deviations from the mean.

In this study, we define the occurrence of a difference in the observed daylight hours-averaged OH number density from the fitted curve of more than two standard deviations as an OH collapse. Taking this definition, Figure 6 presents the number of OH collapses in each year of the 25 year study period. There is clearly a large degree of variability in the number of OH collapses in each year, varying from zero in the year 2011 to 13 in 2005.

**Figure 6:**
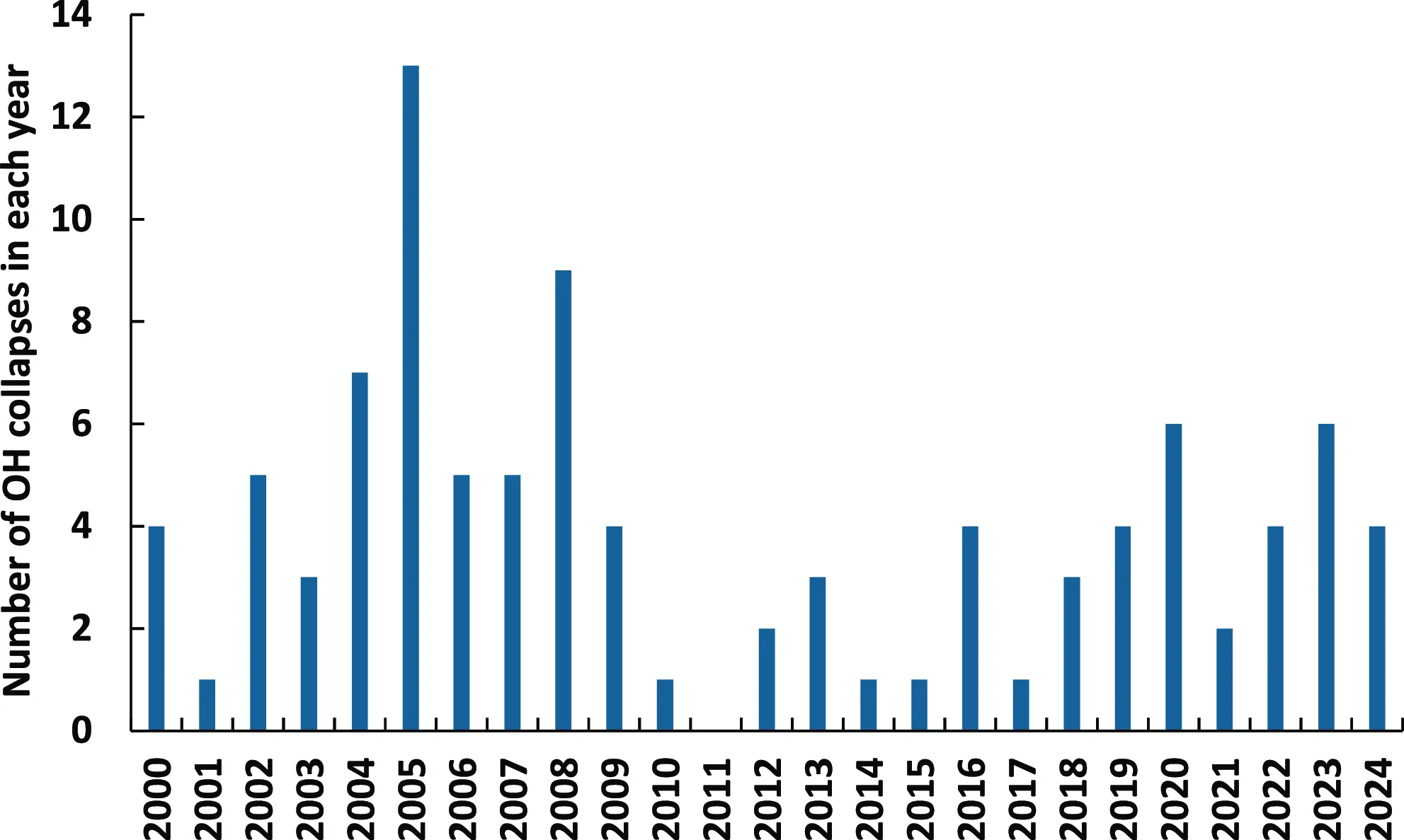
Number of OH collapses in each year of the 25 year study period.

The criterion used to identify OH collapses is entirely arbitrary and has a direct impact on the number of collapses and their distribution during the years of the study period. If the criterion is set too low, then the number of collapses appears huge and the distribution between the years of the study period merely resembles that of the bias in the fitting procedure. Two standard deviations represent a reasonable compromise, producing a number of OH collapses that is illustrative of a potential impact of the impacts of air quality changes on the oxidizing capacity of the London atmosphere.

The London Congestion Charge Scheme (CCS) began on 17th February 2003. Marylebone Road was not actually in the charging zone but formed part of its northern boundary. Traffic flows along the Marylebone Road were markedly influenced before and during the implementation of the CCS as a result of road works to install and change bus lanes, changes to traffic light timings and increased frequency of bus journeys, in addition to the impacts of the CCS itself. The CCS brought about an immediate 15–20% reduction in traffic, with a consequential reduction in vehicle emissions. However, the CCS brought about an increase in harmful NO_2_ reflecting the disproportionate increase in diesel vehicles, such as buses and taxis, with the latter exempt from the CCS charges. The extra buses were fitted with vehicle after-treatment to aid control of diesel particulate emissions. This after-treatment required the production of additional NO_2_ in the vehicle exhaust which is used to oxidize carbon monoxide and hydrocarbons and hence reduce diesel particulate formation. This had the harmful side-effect of increasing dramatically the fraction of NO_*x*_ emitted as NO_2_ by the diesel-engined buses using the Marylebone Road.^52–55^ It is possible that the rise in the number of OH collapses during the early years of the study period shown in Figure 6 has been driven by the increased direct emissions of NO_2_ from diesel-engined vehicles. In this study, the highest numbers of OH collapses were found during 2005 (13 collapses) and 2008 (9 collapses), which can be explained by the higher mixing ratios of NO_2_ (112 ppb during 2005 and 115 ppb during 2008) and higher fraction of NO_*x*_ in the form of NO_2_ (0.38 during 2005 and 0.37 during 2008) (Supporting Information, Figure S1). Although NO_2_ mixing ratios in London Marylebone Road have declined in recent years, the fraction of NO_*x*_ in the form of NO_2_ has increased significantly during 2019–2024 (see Figure S1), which can be responsible for the increase in OH collapses in recent years (Figure 6).

## 4 Conclusions

In the study, we explore the trends and variability of the daylight hours-averaged OH number densities estimated in London Marylebone Road during 2000–2024 using an observations-based approach. Results show a statistically significant downward trend daylight hours-averaged OH number densities of (0.3 ± 0.07) % per decade. The variability in the OH number densities is observed likely because of the changes in emission composition and meteorological effects. The number of OH collapses are found to be highly variable ranging from zero to 13 throughout the years from 2000 to 2024. We find that the AOC in London Marylebone Road site is controlled by NO_*x*_ and NO_2_; however, it could have considerable influence of HCHO photolysis. HONO is known to be the major source of primary OH in London, and the observations-based approach estimates this as a fraction of NO_*x*_, and only HCHO from CH_4_ is considered, with HCHO being an important OH sink and source of HO_2_. The results have lots of potential errors as the OH concentration was only calculated through a simple steady-state equation. The estimation of [HONO] was based on the NO_*x*_ measurement data, which needs London-centric improvement. The major source of HONO is diesel-engined motor vehicles^56^ which have not seen much NO_*x*_ control until very recently. The tropospheric background [HCHO] is a bare minimum because of only considering CH_4_ data from central London for its estimation and this also needs some London-centric improvement. The major source of HCHO is petrol-engined motor vehicles, and these have seen massive reductions in emissions due to the widespread adoption of three-way catalysts in recent years.^57^ All of these approximations for calculating [OH] led to some oversimplification of atmospheric behavior and as such some of the results and explanations in this study may not be representative of a real atmosphere. Furthermore, no secondary chemistry and many OH sources and sinks were not included, including the oxidation of nonmethane organic compounds, which limits the accuracy of the calculations. Overall, it does not seem necessary to completely disregard these findings, despite the many limitations, as numerous studies carry similar unknowns. Instead, this study should be taken as an introduction to the possibility of OH collapse. Further studies in London Marylebone Road and additional urban and rural sites that implement 0-*d* box models, including secondary chemistry and meteorological effects, should be undertaken to assess the likelihood and impact of the findings proposed in this study.

## Supplementary Material



## Notes

Estimates of the yield of HCHO and NO_2_ mixing ratios (PDF)
