# Supplementary material for: Observations-Based Approach to the Estimation of the Oxidizing Capacity of the Atmosphere of Central London over the 2000–2024 Period
Source: J Phys Chem A. 2026 Sep 1;130(37):7450–9. doi: 10.1021/acs.jpca.6c02946 (PMC13592065; doi:10.1021/acs.jpca.6c02946)
Supplement: Supplementary file 1 [file jp6c02946_si_001.pdf]

Supporting Information

Observations-based approach to the estimation of the oxidising capacity of the atmosphere of Central London over the 2000 – 2024 period

M. Anwar H. Khan[a,*], Richard G. Derwent[b], Frank A.F. Winiberg[c], Carl J Percival[c], Dudley E. Shallcross[a,d]

[a]Atmospheric Chemistry Research Group, School of Chemistry, University of Bristol, Cantock's Close, Bristol BS8 1TS, United Kingdom.

[b]rdscientific, Newbury, Berkshire RG14 6LH, United Kingdom.

[c]Jet Propulsion Laboratory, California Institute of Technology, 4800 Oak Grove Dr, Pasadena, CA 91109, USA

[d]Department of Chemistry, University of Western Cape, Robert Sobukwe Road, Bellville, 7535 South Africa.

*Correspondence to M Anwar H Khan, E-mail: anwar.khan@bristol.ac.uk

1. Estimate for the Underestimation of HCHO Mixing Ratios Calculation

The production of HCHO from the oxidation of VOCs has been the subject of several studies and the most recent provide consistent data on the yield of HCHO.[1,2] It emerges that methane not only has a high HCHO yield, but given its very high concentration in the atmosphere, it is the biggest single source globally.[1,2,3] Data concerning VOCs in London are limited but taking the data available we can estimate the production of HCHO over the course of a year and compare these with methane. These data are summarised in Table S1. Derwent and Jenkin calculated the amount of H_2 generated from HCHO photolysis from each compound in Table S1. Noting that the ratio of $\Phi(HO_x)$: $\Phi(H_2)$ they determined was 0.61:0.39, their H_2 yields are converted into HO_x yields instead and these have been used to determine the fraction of HCHO forming HO_x from one molecule of the compound in question. The amount of VOC oxidised in a day is calculated using the given lifetimes and this is then scaled up to a year to provide and estimate of the amount of HCHO generated per year (ppb). Inspection of Table S1 suggests that methane, as expected, dominates (~66%), with isoprene (~ 11%) and methanol (9%) making notable contributions.

Table S1. Estimates of the yield of HCHO (HO_x forming channel) for a variety of measured VOCs in London.

Compound	Lifetime$^{\xi}$	Concentration (ppb)$^{\Upsilon}$	HCHO yield	Estimated HCHO produced yr^{-1}/ppb	% contribution
Methane	9.1 years	2800	0.61	291	66.4
Methanol	15 days	5	0.33	38.7	8.81
Isoprene	0.125 days	0.1	1.29	47.4	10.8
Acetone	30 days	1	0.78	0.02	2.1
Ethane	15 days	1	0.20	0.01	1.1
Ethene	1.4 days	0.1	0.82	15.4	3.5
Propane	10 days	0.38	0.02	0.2	0.0
Propene	0.25 days	0.06	0.89	19.2	4.4
isobutane	4.7 days	0.1	0.39	2.7	0.6
n-butane	4.7 days	0.25	0.17	3.0	0.7
isopentane	4.0 days	0.1	0.20	1.6	0.4
n-pentane	4.0 days	0.07	0.31	1.8	0.4
Benzene	9.4 days	0.04	0.11	0.2	0.0

Toluene	1.9 days	0.05	0.38	2.8	0.6

data sources: ξMethane's lifetime (Staniaszek et al.[4]) and ξThe other VOCs lifetimes (Atkinson[5]), ϒMethane's concentration (Saboya et al.[6]), ϒMethanol's concentration (Valach et al.[7]), ϒOther VOCs concentrations (Ashworth et al.[8]).

2. The mixing ratios of NO2 and the fraction of NO_x in the form of NO_2 for the period of 2020-2024

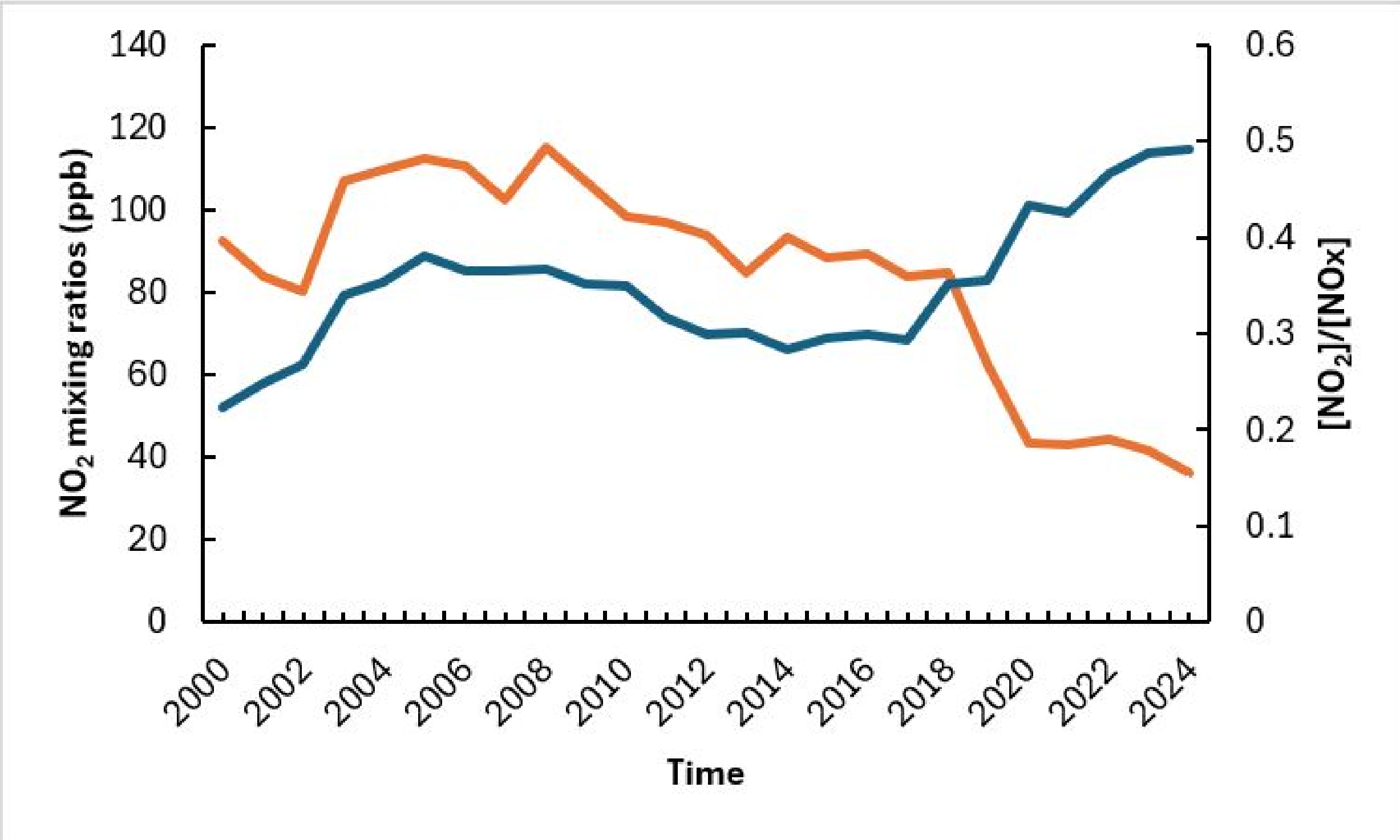

Figure S1: NO_2 mixing ratios and the fraction of NO_x in the form of NO_2 for the period of 2020-2024

References

1. Derwent, R. G.; Jenkin, M. E.; Stevenson, D. S.; Utembe, S.R.; Khan, M. A. H.; Shallcross, D. E. Influence of the oxidation of non-methane volatile organic compounds on tropospheric hydrogen: A STOCHEM-CRI global Lagrangian model study, *Atmos. Environ.* **2025**, *352*, 121214.

2. Derwent, R. G.; Jenkin, M. E. Estimation of the atmospheric hydrogen source from the oxidation of man-made and natural non-methane organic compounds using a Master Chemical Mechanism. *Atmos. Environ.* **2024**, *339*, 120871.

3. Paulot, F.; Paynter, D.; Naik, V. ; Malyshev, S. ; Menzel, R.; Horowitz, L. W. Global modeling of hydrogen using GFDL-AM4.1: Sensitivity of soil removal and radiative forcing. *Int. J. Hydrogen Energy* **2021**, *46*, 13446-13460.

4. Staniaszek, Z.; Griffiths, P.T.; Folberth, G.A.; O'Connor, F. M.; Abraham, N. L.; Archibald, A. T. The role of future anthropogenic methane emissions in air quality and climate. *npj Clim. Atmos. Sci.* **2022**, *5*, 21.

5. Atkinson, R. Atmospheric chemistry of VOCs and NO_x. *Atmos. Environ.* **2000**, *34*, 2063-2101.

6. Saboya, E.; Zazzeri, G.; Graven, H.; Manning, A. J.; Englund Michel, S. London measurements: Continuous CH_4 and $\delta^{13}CH_4$ measurements in London demonstrate under-reported natural gas leakage. *Atmos. Chem. Phys.*, **2022**, *22*, 3595–3613.

7. Valach, A.C.; Langford, B.; Nemitz, E.; MacKenzie, A. R.; Hewitt, C. N. Concentrations of selected volatile organic compounds at kerbside and background sites in central London, *Atmos. Environ.*, **2014**, *95*, 456-467.

8. Ashworth, K.; Bucci, S.; Gallimore, P. J.; Lee, J.; Nelson, B. S.; Sanchez-Marroquín, A.; Schimp, M. B.; Smith, P. D.; Drysdale, W. S.; Hopkins, J. R.; Lee, J. D.; Pitt, J. R.; Di Carlo, P.; Krejci, R.; McQuaid, J. B. Megacity and local contributions to regional air pollution: an aircraft case study over London. *Atmos. Chem. Phys.*, **2020**, *20*, 7193–7216.
